# Evaluation of the Therapeutic Use of Local Anesthetics in Chronic Myogenous Temporomandibular Disorders: A Pilot Study and Evidence Gap Assessment

**DOI:** 10.3390/jcm15208112

**Published:** 2026-10-19

**Authors:** Güzin Neda Hasanoğlu Erbaşar, Onur Odabaşı, Sercan Dolanbay, Kevser Tütüncüler

**Affiliations:** 1 Department of Oral and Maxillofacial Surgery, Faculty of Dentistry, Ankara Yıldırım Beyazıt University, 06010 Ankara, Turkey; 2 Department of Oral Health & Medicine, University Center of Dental Medicine Basel, University of Basel, 4058 Basel, Switzerland

**Keywords:** temporomandibular disorders, myalgia, neural therapy, therapeutic local anesthetic, orofacial pain

## Abstract

**Background/Objectives:** The therapeutic use of local anesthetics has been discussed in several musculoskeletal pain conditions, but its role in myogenous temporomandibular disorders (TMDs) remains unclear. This study evaluated clinical outcomes after a therapeutic local anesthetic injection protocol in patients with chronic myogenous TMDs and examined whether comparable clinical evidence exists in the literature. **Methods:** This retrospective study included patients diagnosed with myalgia according to the Diagnostic Criteria for Temporomandibular Disorders (DC/TMD) criteria. Patients received six weekly sessions of therapeutic local anesthetic injections targeting trigeminal nerve exit points, myofascial trigger points in the masticatory and cervical muscles, the projection area of the otic ganglion, and upper cervical segments. Mandibular mobility, pain intensity, and DC/TMD Axis II measures were compared between baseline and 3-month follow-up. A structured literature search was also performed in PubMed, Cochrane Library, LIVIVO, Ovid MEDLINE, Scopus, and Web of Science. **Results:** Twenty-seven patients were included. Pain-free mouth opening, maximum unassisted mouth opening, and maximum assisted mouth opening increased after therapy. Pain intensity and patient-reported measures of jaw function, depressive and anxiety symptom scores, somatic symptom scores, and oral behavior scores decreased. The literature search identified 1406 records; after duplicate removal, 561 records were screened. Several studies investigating local injections for TMDs were identified; however, none appeared to evaluate the specific injection protocol used in this cohort. **Conclusions:** Therapeutic local anesthetic injections were associated with improvements in pain, jaw function, and patient-reported outcomes in this retrospective pilot cohort. Owing to the lack of a control group, the findings should be interpreted with caution.

## 1. Introduction

Temporomandibular disorders (TMDs) comprise a heterogeneous group of musculoskeletal conditions involving the temporomandibular joint (TMJ), masticatory muscles, and associated tissues [1]. TMDs are the second most common musculoskeletal condition after chronic low back pain [2]. This condition affects approximately one-third of the population, and more than half of the affected individuals have pain [3]. Most of the cases can resolve spontaneously, a portion can show recurrence, and approximately 15% progress to chronic TMDs [4]. The mechanisms underlying this chronification process, however, have not been clearly demonstrated. Nevertheless, it has been suggested that psychosocial factors, peripheral nociceptive input, and altered central pain processing may contribute to the chronification of symptoms in TMD patients [5].

Considering the biopsychosocial constructs involved in the etiology of TMDs, psychosocial evaluation is considered as important as a careful and standardized clinical assessment in establishing an accurate differential diagnosis [6]. For this purpose, the Diagnostic Criteria for Temporomandibular Disorders (DC/TMD) has become one of the most widely used diagnostic tools in orofacial pain practice. Through its dual-axis structure, this protocol not only provides a physical diagnosis of pain-related or intra-articular TMDs based on anamnesis and clinical examination, but also assesses psychosocial status and pain-related disability using validated self-report instruments [7].

Current consensus statements and clinical guidelines recommend that therapy for TMDs should begin primarily with conservative and reversible approaches. This approach generally includes pharmacotherapy, cognitive-behavioral therapy, physiotherapy, and the short-term use of intraoral appliances; the main goal is to establish a therapy process that is tailored to the patient’s needs, as safe as possible, and sustainable over time [6,8]. Additionally, minimally invasive needling-based therapies are increasingly utilized, particularly in patients with myogenous TMDs. These approaches may involve either dry needling alone or injections with pharmacological substances such as botulinum toxin and local anesthetics (LA) [9]. The therapeutic use of LA [10,11,12] represents one injection-based approach used in various musculoskeletal and chronic pain conditions. Here, “therapeutic” refers to the intended clinical application of the injections; in German-speaking countries, this approach is referred to as neural therapy (NT). It has gradually expanded worldwide and is currently included in the mandatory basic health insurance systems of several countries, including Switzerland, Austria, and Colombia [13]. This therapeutic approach typically involves the administration of low doses of short-acting LA such as procaine or lidocaine into areas of nerve exit sites, ganglia, and myofascial trigger points or tender points, aiming to interrupt persistent peripheral nociceptive input and modulate autonomic regulation and neuroplastic processes within the nervous system, particularly the autonomic nervous system (ANS) [13].

Although the therapeutic use of LA has been well documented in several musculoskeletal pain disorders, data regarding its role in orofacial pain remains scarce. The aim of this retrospective study was to evaluate clinical outcomes associated with therapeutic use of LA in patients with chronic myogenous TMDs and to assess changes in pain levels and functional outcomes from baseline to the 3-month follow-up. Additionally, a structured literature search was conducted to identify existing evidence gaps in this field.

## 2. Materials and Methods

### 2.1. Study Design and Patient Selection

This retrospective study evaluated patients diagnosed with myalgia according to the DC/TMD criteria who underwent therapeutic LA injections at the Ankara Yıldırım Beyazıt University Oral and Maxillofacial Surgery Clinic between October 2023 and October 2024. We excluded patients with a symptom duration of less than 6 months, concomitant intra-articular disorders, a history of TMDs management within the previous three months, or those who received additional therapies combined with LA injections. Further exclusion criteria comprised pregnancy or lactation, a history of neck or back surgery, current antidepressant use at the time of management, intellectual disability, severe cognitive impairment, and absence of a follow-up assessment for the primary outcome.

Because of the retrospective pilot design, no a priori sample size calculation was performed; all patients who met the eligibility criteria during the study period were included.

The injections were administered as part of routine clinical care between October 2023 and October 2024. The subsequent retrospective analysis of the clinical records was approved by the Ankara Yıldırım Beyazıt University Ethics Committee on 21 November 2024 (Decision No. 09/961).

### 2.2. Clinical Assessment

Clinical data were extracted from the Turkish version of the standardized DC/TMD examination forms routinely used in our clinic [14]. Mandibular mobility was evaluated at baseline and at the 3-month follow-up by measuring painless mouth opening, maximum unassisted opening, maximum assisted opening, as well as lateral and protrusive mandibular movements. Pain intensity was rated using a 10-cm visual analog scale (VAS). To assess psychosocial status and pain-related disability, we used standardized DC/TMD Axis II instruments, including the Graded Chronic Pain Scale (GCPS), Jaw Functional Limitation Scale-8 (JFLS-8), Patient Health Questionnaire-9 (PHQ-9) [15], Generalized Anxiety Disorder-7 (GAD-7), Patient Health Questionnaire-15 (PHQ-15), and Oral Behaviors Checklist (OBC). Specifically, chronic pain severity and its impact on daily functioning were evaluated via the GCPS, which classifies patients into five distinct levels ranging from Grade 0 (no pain) to Grade IV (high disability and severe limitation). Available clinical records were also reviewed for procedure-related adverse events. Potential adverse events were identified from existing visit notes. The available records did not document a predefined complication checklist or routine questioning about adverse events.

### 2.3. Therapeutic Local Anesthetic Procedure

All injections were administered by the same clinician (G.N.H.E.), while outcome data were collected by a separate investigator (K.T.). Procaine hydrochloride (NTCAIN® 100 mg/10 mL, Assos Pharmaceuticals, Istanbul, Türkiye) was diluted 1:1 with sterile 0.9% sodium chloride solution (TURKFLEKS, TURK İlaç ve Serum Sanayi A.Ş., Ankara, Türkiye) to obtain a final concentration of 0.5% (5 mg/mL). Injection sites were identified by clinical examination and palpation. No imaging guidance was used. No topical anesthetic was applied before the injections. The 26-gauge, 13-mm and 27-gauge, 40-mm hypodermic needles used were SET needles (Set Medikal San. ve Tic. A.Ş., Istanbul, Türkiye).

At each session, all patients received bilateral injections at the supraorbital, infraorbital, and mental nerve exit-point regions (0.5 mL per site; six injections, 3.0 mL in total). The supraorbital injections were performed using a 26-gauge, 13-mm needle, whereas the infraorbital and mental injections were performed via an intraoral approach using a 27-gauge, 40-mm needle. Bilateral injections were also administered in the anatomical projection area of the otic ganglion (2.5 mL per side; two injections, 5.0 mL in total). For these injections, the entry point was identified by palpation in the region of the mandibular notch, approximately 3 cm anterior to the tragus and immediately inferior to the zygomatic arch. A 27-gauge, 40-mm needle was introduced from the lateral aspect and advanced approximately 4 cm with a slight cranial and posterior angulation. According to the treating clinician, aspiration was routinely performed before procaine injection at these sites to check for blood return and reduce the risk of inadvertent intravascular injection. Because needle-tip position was not confirmed by imaging, these injections are described as being performed in the anatomical projection area of the otic ganglion rather than within the ganglion itself.

In the cervical region, one intradermal wheal was placed approximately 2 cm lateral to the spinous process on each side at each of the C2, C3, and C4 levels (0.2 mL per site; six injections, 1.2 mL in total). A 26-gauge, 13-mm needle was inserted superficially into the skin at an angle of approximately 10–30° to create each wheal.

Additional injections were administered to myofascial trigger points in the temporalis, masseter, sternocleidomastoid, and trapezius muscles when identified by palpation. A taut band was first located in the muscle, followed by the most tender point within that band. Injection sites were selected individually at each session rather than being predetermined anatomical sites. Each identified trigger point received 0.2 mL of 0.5% procaine. A 27-gauge, 40-mm needle was used for trapezius injections, and a 26-gauge, 13-mm needle was used for injections into the other muscles. The number of additional trigger point injections varied among patients and sessions but was not documented in the patient records. Therefore, the distribution of additional injection counts and the total injected volume and procaine dose per session could not be reconstructed retrospectively.

The fixed components of the protocol comprised 14 injection sites and 9.2 mL of 0.5% procaine (46 mg) per session. The total procaine dose per session, including both fixed-site injections and additional myofascial trigger point injections, was limited to 6 mg/kg of body weight. The fixed injection sites and the muscles in which additional myofascial trigger point injections were administered are illustrated in Supplementary Figure S1.

Baseline assessments were performed one week before the first treatment session. Patients then received six sessions, once weekly during weeks 1–6, and were reassessed six weeks after the final session (week 12), approximately three months after baseline. Patients who received documented additional therapies for TMDs in combination with the injection protocol were excluded from the analysis, as described in the participant flow. Rescue analgesics were not prohibited during follow-up; their use was not systematically recorded in the clinical records.

### 2.4. Literature Search

Electronic database searches were conducted across PubMed, Cochrane Library, LIVIVO, Ovid MEDLINE, Scopus, and Web of Science. The final search was performed on 20 June 2026. The search strategy combined terms related to TMDs, orofacial pain, myofascial pain, myalgia, and masticatory muscle pain with terms related to lidocaine, procaine, neural therapy, and therapeutic LA injections. Medical Subject Headings (MeSH) were utilized in PubMed where appropriate, and the search syntax was tailored to the specific indexing structure of each database. The complete database-specific search strategies and the number of records retrieved from each platform are detailed in Supplementary Table S1.

After all records were combined, they were exported to Microsoft Excel for Mac, version 16.112.1 (Microsoft Corporation, Redmond, WA, USA). Duplicates were identified using title, authors, publication year, and DOI when available, and were then manually checked before removal. The remaining unique records were screened at the title and abstract levels using a predefined four-step selection and classification process. Title and abstract screening was performed independently by two reviewers (O.O. and S.D.). Disagreements and uncertain records were resolved through discussion with a third reviewer (G.N.H.E.). In the first step, records were assessed for basic eligibility. Systematic reviews, narrative reviews, meta-analyses, protocols, animal studies, non-clinical studies, and publications in languages other than English or Turkish were excluded. The language restriction reflected the languages in which the reviewers could reliably assess the reports.

In the second step, records unrelated to the target clinical question were excluded. These included studies on general or procedural anesthesia; studies on body or generalized myofascial pain syndrome outside the orofacial region; and studies on non-target orofacial pain conditions, such as neuropathic or odontogenic pain conditions not related to TMDs or orofacial myogenous pain.

In the third step, records that passed the first two steps and were related to TMDs or orofacial pain were classified according to article type as research articles or case reports/case series. No record was excluded at this stage. In the fourth step, the remaining studies were classified according to the intervention type.

For the final intervention comparability assessment, we used a predefined intervention framework based on the therapeutic use of LA. Within this framework, directly comparable interventions were expected to involve low-dose, short-acting LA, such as procaine or lidocaine, administered to neuroanatomically or neuroregulatorily relevant targets, including nerve exit points, ganglia, segmental intradermal wheal/quaddel areas, and autonomic or segmental targets. Studies using LA only for procedural anesthesia, as a carrier or comparator, in intra-articular joint-directed therapy, or as isolated myofascial trigger point injections were considered related but not directly comparable with the protocol evaluated in the present study. Because isolated myofascial trigger point injections differ conceptually from neural therapy-oriented protocols, they were documented as related interventions rather than as direct evidence. Full texts were sought when eligibility could not be determined from the title and abstract. When a full text was unavailable and the title and abstract provided insufficient information, intervention comparability was recorded as uncertain. A formal risk-of-bias assessment and quantitative synthesis were not performed; the literature findings were summarized descriptively. The descriptive summary was used to place the present clinical findings in the context of related injection studies.

### 2.5. Statistical Analysis

Data analysis was performed using IBM SPSS Statistics version 25.0 (IBM Corp., Armonk, NY, USA). Categorical variables were presented as numbers and percentages. Continuous variables were reported as mean ± standard deviation or median [interquartile range], depending on their distributional characteristics. The normality of paired differences between baseline and follow-up measurements was assessed using the Shapiro–Wilk test.

Baseline and follow-up values were compared using paired statistical tests. For each outcome, only patients with both baseline and follow-up measurements were included in the paired analysis; missing measurements were not imputed. Normally distributed paired continuous variables were analyzed using the paired samples *t*-test. Non-normally distributed continuous variables and ordinal outcomes were analyzed using the Wilcoxon signed-rank test. For VAS, JFLS-8, PHQ-9, GAD-7, PHQ-15, and OBC, the 95% confidence interval for the median paired change was calculated from 10,000 patient-level bootstrap samples drawn with replacement; the 2.5th and 97.5th percentiles of the bootstrap estimates defined the interval. Bootstrap confidence intervals were calculated using Python version 3.12.14 with NumPy version 2.3.5. Changes in GCPS grades and pain-related disability categories were also assessed with the Wilcoxon signed-rank test. Pain intensity measured by VAS was designated as the primary clinical outcome, whereas mandibular mobility and DC/TMD Axis II measures were treated as secondary exploratory outcomes. Given the retrospective pilot design and the absence of a control group, the statistical analyses were intended to be exploratory rather than confirmatory. A *p* value < 0.05 was considered statistically significant. No adjustment for multiple comparisons was applied; findings for secondary outcomes were therefore interpreted as exploratory.

## 3. Results

A total of 70 patients diagnosed with myalgia according to the DC/TMD criteria were initially identified and screened for eligibility. Of these, 11 patients were excluded because of concomitant intra-articular disorders, 10 because they had received additional therapies combined with therapeutic LA injections, 7 because they had received therapies for TMDs within the previous three months, 5 because of antidepressant use, 4 because of symptom durations of less than 6 months, and 6 because they did not attend follow-up visits. Consequently, 27 patients were included in the final analysis. The mean age was 33.1 ± 10.8 years (range: 18–56 years), and the majority of included patients were female (*n* = 22, 81.5%). The median symptom duration was 36 months (range: 6–240 months) and the median baseline pain intensity was 6.0 [4.0–7.0] (Table 1). Because some outcome-specific data were unavailable, the number of patients included in individual analyses varied according to data availability (Table 1, Table 2 and Table 3).

Table 2 presents the comparisons of mandibular movements before and after therapeutic LA injections. Significant increases were observed in pain-free mouth opening, maximum unassisted mouth opening, and maximum assisted mouth opening following therapy (*p* < 0.001, *p* = 0.012, and *p* = 0.015, respectively). The corresponding mean paired changes were 7.89 mm (95% CI, 4.56–11.21), 3.22 mm (95% CI, 0.77–5.66), and 2.58 mm (95% CI, 0.55–4.61), respectively. In contrast, no significant changes were observed in lateral or protrusive movements (*p* > 0.05) (Table 2).

Table 3 presents the scores of psychosocial status and pain-related disability of patients before and after therapeutic LA injections. Statistically significant reductions were observed in VAS, JFLS-8, PHQ-9, GAD-7, PHQ-15, and OBC scores (*p* < 0.05) (Table 3). The median paired VAS change was −3.0 points (95% CI, −5.0 to −2.0). For JFLS-8, 20 patients had no paired change and seven had lower scores at follow-up. The median paired change was therefore 0.00 (95% CI, 0.0 to 0.0), while the Wilcoxon signed-rank test based on nonzero differences yielded *p* = 0.018.

GCPS grades and pain-related disability categories also improved after therapy. The proportion of patients classified as having no chronic pain increased from 22.2% at baseline to 66.7% at follow-up, while moderate-to-severe pain-related disability decreased markedly (GCPS grades: *p* = 0.006; pain-related disability categories: *p* = 0.003). Detailed GCPS distributions are provided in Supplementary Table S4.

Redness was mentioned in one treatment record. Its duration and relationship to the injection were not documented.

### Results of the Literature Search and Evidence Gap Assessment

The database search yielded 1406 records in total: 285 from PubMed, 216 from Cochrane Library, 291 from LIVIVO, 270 from Ovid MEDLINE, 188 from Scopus, and 156 from Web of Science. After removal of 845 duplicate records, 561 unique records were screened by title and abstract.

In the first screening step, 165 records were excluded because they did not meet the basic eligibility criteria. These included 78 protocol records; 47 reviews, systematic reviews or meta-analyses; 22 animal studies; 14 publications in languages other than English or Turkish; and 4 non-clinical studies. The remaining 396 records were evaluated for clinical relevance to the target topic.

In the second step, 340 records were excluded as unrelated to the target clinical question. Of these, 77 were related to general or procedural anesthesia, 170 addressed body or generalized myofascial pain syndrome outside the orofacial region, and 93 evaluated other orofacial pain conditions not directly related to TMDs or orofacial myogenous pain. After this step, 56 records remained for further classification.

In the third step, the 56 records related to TMDs or orofacial pain were classified according to article type. Forty-nine were research reports, and seven were case reports or case series. No record was excluded at this stage. In the fourth step, 18 records were classified as having interventions outside the present framework. Intervention details remained insufficient for two further records. The remaining 36 publications were classified as clinically related. These included studies of isolated muscle injections as well as combined injection approaches. Other related interventions included isolated intramuscular LA injection, PRP/PRGF or steroid comparison studies, prolotherapy or dextrose studies, iontophoresis, nerve or periarticular blocks without a neural therapy-oriented protocol, intra-articular corticosteroid/LA injections, and other borderline or unclear interventions. The related publications and their comparability status are provided in Supplementary Table S2. The intervention categories and reasons for non-comparability or uncertainty are summarized in Supplementary Table S3.

Two particularly borderline studies were reviewed in full text. One involved local injection therapy for myofascial pain syndrome of the head and neck; however, the intervention was limited to trigger point/local infiltration therapy rather than a neural therapy-oriented protocol involving neuroanatomical, segmental, or autonomic targets. The other was a case report involving combined trigger point injection and stellate ganglion block for non-odontogenic mandibular molar pain referred from the masseter muscle. Although this case included an autonomic block, it was not a direct myogenous TMDs study and did not use a neural therapy-oriented protocol comparable with the present intervention. Therefore, neither study was considered directly comparable with the present protocol. An additional full-text case report, listed in Supplementary Table S2, described trigger point injections together with an infraorbital lidocaine block for facial myofascial pain; it did not evaluate the injection protocol used in the present cohort.

**Figure 1:**
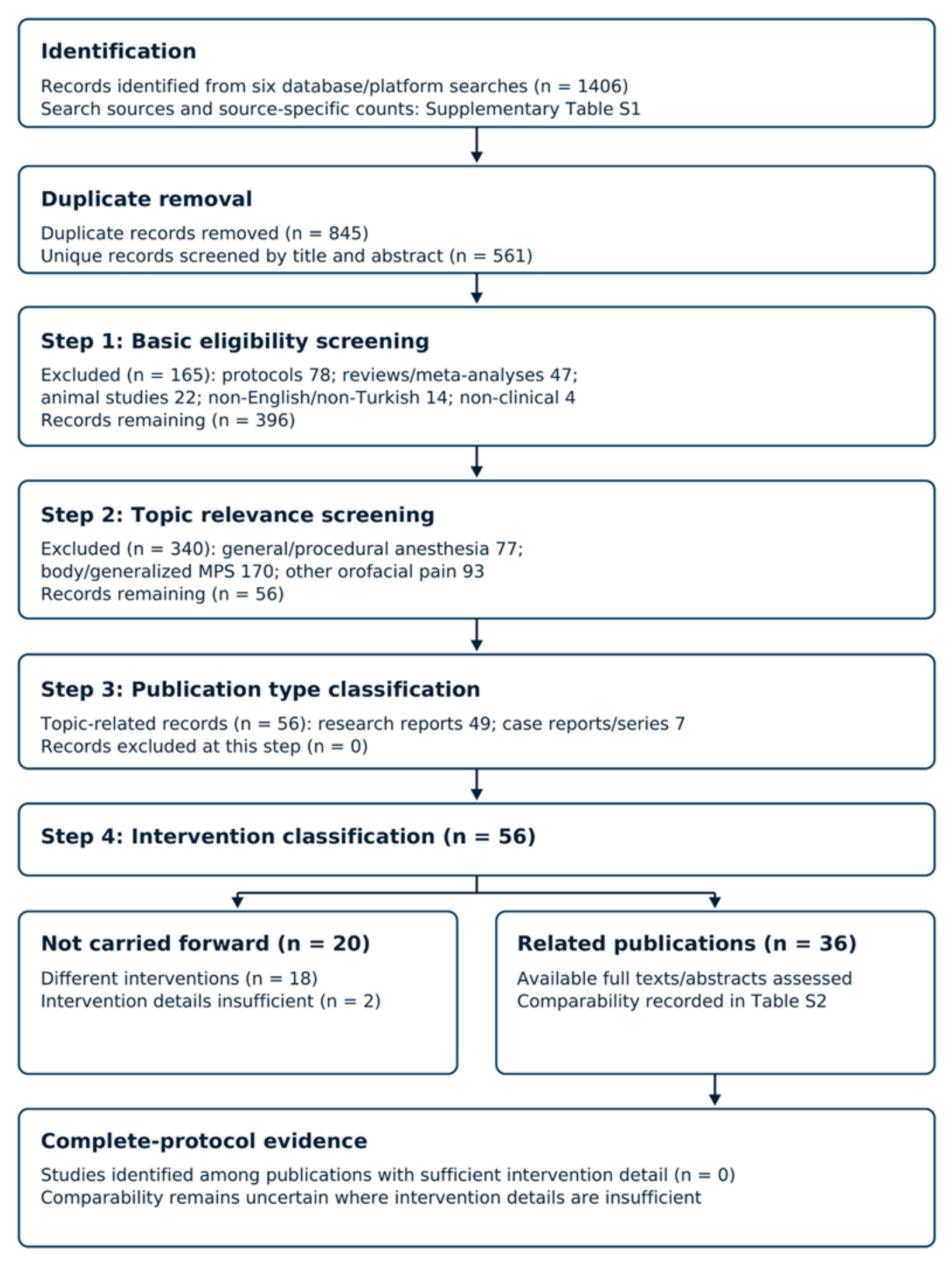
Structured literature search and record classification flow. LA, local anesthetic; MPS, myofascial pain syndrome; TMD, temporomandibular disorder.

Overall, no study of the complete protocol used in the present cohort could be confirmed among reports with sufficient intervention details; comparability remained uncertain for reports with insufficient information. The structured literature search and record classification are shown in Figure 1.

## 4. Discussion

In the present study, patients with chronic myogenous TMDs who received six sessions of therapeutic LA injections showed improvements in mandibular mobility capacity at the follow-up assessment performed 6 weeks after completion of sessions. The mean change was larger for pain-free mouth opening (7.89 mm) than for maximum unassisted (3.22 mm) or assisted opening (2.58 mm). A TMD-specific study reported varying estimates for clinically important changes in orofacial pain and an estimate of approximately 2.5 mm for maximum mouth opening [16]. These external estimates do not establish how many patients in the present cohort achieved a clinically important improvement. In addition to the reduction in pain intensity, this therapeutic approach was associated with significant improvements in chronic pain-related disability, as demonstrated by the shift toward lower GCPS grades and less disabling chronic pain categories. Although the literature search did not identify a study of the complete protocol used here among reports with sufficient intervention details, the observed improvements are broadly consistent with findings reported in studies evaluating the therapeutic use of LA in other chronic pain conditions [10,11,12].

Several mechanisms may provide a rationale for therapeutic LA injections in chronic myogenous TMDs; however, their contribution to the observed clinical outcomes could not be assessed in the current study. Mechanical overload, ischemia, or inflammation in the masticatory system may activate peripheral nociceptors and trigger the release of excitatory neurotransmitters and neuropeptides. These mediators promote neurogenic inflammation and peripheral sensitization by lowering nociceptor activation thresholds, increasing excitability, and recruiting previously silent nociceptors [10,17]. Persistent nociceptive input may also contribute to central sensitization through altered trigeminal nociceptive processing and impaired descending inhibitory control [17,18]. Interactions between afferent neurons and satellite glial cells within the trigeminal ganglion may further enhance sensitization through the release of inflammatory mediators [19,20]. LA have reported anti-inflammatory properties involving the inhibition of multiple inflammatory cascades [21]. Repeated administration has therefore been proposed to attenuate neurogenic inflammation and interrupt self-perpetuating pain cycles in chronic pain conditions [10]. Another proposed mechanism involves modulation of autonomic and neuroimmune pathways. LA injections at selected sites may interrupt sympathetic–afferent reflex circuits hypothesized to sustain inflammation and sensitization [10,22].

Autonomic dysfunction reported in patients with TMDs suggests a possible autonomic nervous system contribution to pain persistence [23,24]. The close anatomical relationship between sympathetic efferent fibers and nociceptive afferents in the TMJ and masticatory muscles may modulate somatosensory processing, sensitization, and neuroinflammation at the peripheral level [23]. Trigeminal–autonomic interactions have also been described in the brainstem and upper cervical spinal cord [22,25]. These interactions may influence pain modulation within central trigeminal networks, since the spinal trigeminal nucleus extends from the medulla into the upper cervical segments [26]. Consequently, in addition to the other injection sites, the upper cervical region was selected as a potential neuromodulatory target due to its close functional integration with trigeminal and autonomic pathways.

Whether repeated injections affect these pathways or account for effects beyond the anesthetic period remains unknown. The literature search identified related LA interventions, including muscle injections and approaches combining muscle injections with nerve blocks. These reports provide evidence relevant to individual components, but they do not establish the effects of the combined protocol used in the present cohort. This distinction is important because isolated trigger point injection should not be considered equivalent to neural therapy. In the present study, the therapeutic LA protocol included myofascial trigger point injections in the masticatory and cervical muscles, as well as injections at trigeminal nerve exit points, the otic ganglion projection area, and upper cervical segments using intradermal wheals.

It must be acknowledged that this pilot study is not without limitations. Spontaneous improvement of signs and symptoms of TMDs has been observed in a subset of patients [27,28]. In an observational study of patients with low pain-related impairment, muscle-disorder diagnoses decreased from 68.1% at baseline to 23.1% after 2–3 years [29]. Because that study involved initial counselling, a different outcome, and a much longer follow-up, these figures cannot estimate the untreated 12-week change in our patients. Furthermore, given the fluctuating nature of TMDs, the phenomenon of regression to the mean may also contribute to symptom improvement in some individuals [27,29]. In the absence of a control group, it is not possible to estimate the extent to which spontaneous remission or regression to the mean may have influenced the observed outcomes. Rescue analgesic use during follow-up could not be reconstructed from the available records; therefore, its possible contribution to the observed change in VAS scores could not be assessed. Consequently, the specific therapeutic effect of LA injections cannot be determined with certainty. The patients included in the present study represented chronic cases, with a median symptom duration of 36 months; nevertheless, chronicity of symptoms does not exclude spontaneous improvement or regression to the mean. Furthermore, as with any therapeutic intervention, contextual and placebo-related effects, including clinician–patient interactions and patients’ expectations and perceptions, may have influenced the observed outcomes [27,29]. Additionally, findings from the placebo literature suggest that more invasive procedures tend to show larger placebo effects [30]. Therefore, when interpreting the results of an invasive procedure like therapeutic LA injections, the potential contribution of these effects should also be considered. Changes in psychosocial scores cannot be attributed specifically to procaine. Pain and psychosocial measures were assessed at the same two time points; the temporal or causal relationship between their changes could not be determined. Excluding patients who used antidepressants may also limit the generalizability of these findings. Previous TMD treatment, comorbidity, and medication histories were not recorded in a standardized manner. These characteristics could therefore not be summarized reliably for the included patients. Because adverse events were assessed retrospectively without standardized monitoring, underreporting is possible, and these records cannot establish the safety of the procedure. The sample size was determined by available eligible records rather than an a priori calculation; with 27 patients, effect estimates are imprecise and uncommon adverse events may not be captured. Some secondary mandibular movement measurements were unavailable at follow-up, leaving 24 to 26 paired observations for these analyses. The exclusion of six patients who did not attend follow-up may also have introduced selection bias. The search was restricted to English and Turkish because these were the languages available for screening and assessment; relevant publications in other languages, including German, may therefore have been missed. Grey literature was not searched, and no formal risk-of-bias assessment was performed for the related studies.

## 5. Conclusions

In this pilot study, patients with chronic myogenous TMDs, many of whom had experienced symptoms for several years, demonstrated improvements in jaw function, pain intensity, and pain-related disability following the therapeutic LA injection protocol. These before–after observations warrant evaluation in prospective controlled studies. Nevertheless, randomized controlled trials are needed to determine the extent to which the observed improvements can be attributed to the intervention itself rather than to non-specific effects, including spontaneous improvement, regression to the mean, and placebo responses. The accompanying search identified related injection studies but did not confirm a study of the specific combined protocol used here among reports with sufficient intervention details.

## Figures and Tables

**Table 1:** Demographic and clinical characteristics of included cases.

	*n* = 27
Age (years) *	33.1 ± 10.8
Age range (years)	18–56
Gender	
Female	22 (81.5%)
Male	5 (18.5%)
Body weight (kg) *	67.2 ± 11.4
Body height (m) *	1.67 ± 0.082
Body mass index (kg/m^2^) *	24.1 ± 3.7
Duration of symptoms (months) **	36 (6.0–240)
Baseline pain intensity ***	6.0 [4.0–7.0]

Data are shown as * mean ± SD, ** median (min–max), or *** median [25th–75th] percentiles.

**Table 2:** Comparison of mandibular movement measurements before and after therapeutic LA injections.

Mandibular Movement	*n*	Baseline	Follow-Up	*p*-Value †	Mean Change ± SD	95% CI
Pain-free mouth opening (mm)	27	31.27 ± 9.73	39.16 ± 8.15	<0.001	7.89 ± 8.40	4.56, 11.21
Maximum unassisted mouth opening (mm)	27	42.56 ± 8.51	45.78 ± 6.11	0.012	3.22 ± 6.19	0.77, 5.66
Maximum assisted mouth opening (mm)	26	45.59 ± 7.79	48.17 ± 6.17	0.015	2.58 ± 5.03	0.55, 4.61
Right lateral movement (mm)	24	7.95 ± 2.74	8.54 ± 2.76	0.399	0.59 ± 3.37	−0.83, 2.01
Left lateral movement (mm)	24	8.55 ± 2.49	8.59 ± 2.77	0.948	0.04 ± 3.22	−1.32, 1.40
Protrusion level (mm)	24	5.70 ± 2.39	5.78 ± 2.44	0.883	0.08 ± 2.74	−1.08, 1.24

Data are presented as mean ± SD. The *n* for each outcome represents patients with both baseline and follow-up measurements. Paired change was calculated for each patient as follow-up minus baseline. The 95% confidence intervals refer to the mean paired changes. † Paired-samples *t*-test.

**Table 3:** Comparison of patient-reported outcome measures related to psychosocial status and pain-related disability before and after therapeutic LA injections.

Outcome	*n*	Baseline	Follow-Up	*p*-Value †	Median Change [IQR]	95% CI
VAS	27	6.0 [4.0–7.0]	2.0 [1.0–4.0]	<0.001	−3.0 [−5.0–−1.0]	−5.0, −2.0
JFLS-8	27	1.12 [0.60–3.37]	0.62 [0.37–1.50]	0.018	0.00 [−1.27–0.00]	0.0, 0.0
PHQ-9	27	9.0 [5.0–11.0]	3.0 [2.0–8.0]	<0.001	−3.0 [−8.0–−2.0]	−7.0, −2.0
GAD-7	27	8.0 [5.0–11.0]	4.0 [1.0–7.0]	<0.001	−5.0 [−6.0–−3.0]	−5.0, −3.0
PHQ-15	27	10.0 [8.0–15.0]	5.0 [3.0–10.0]	<0.001	−4.0 [−8.0–−1.0]	−7.0, −2.0
OBC	26	30.0 [20.0–40.0]	17.5 [11.0–24.75]	0.002	−12.0 [−22.25–−0.75]	−20.0, −3.0

VAS, visual analog scale; JFLS-8, eight-item Jaw Functional Limitation Scale; PHQ-9, Patient Health Questionnaire-9; GAD-7, Generalized Anxiety Disorder-7; PHQ-15, Patient Health Questionnaire-15; OBC, Oral Behaviors Checklist. Data are presented as median [25th–75th percentiles]. For each outcome, analyses were based on patients with available paired baseline and follow-up data. Paired change was calculated for each patient as follow-up minus baseline. No imputation was performed for missing data. The median paired change may differ from the difference between the baseline and follow-up medians. The 95% confidence intervals refer to the median paired changes and were estimated by bootstrap resampling. † Wilcoxon signed-rank test.

## Data Availability

The data that support the findings of this study are not publicly available due to ethical and privacy restrictions related to retrospective clinical records. Deidentified data may be made available from the corresponding author upon reasonable request and subject to applicable ethical approvals.

## Supplementary Materials

The following supporting information can be downloaded at: https://www.mdpi.com/article/10.3390/jcm15208112/s1: Supplementary Table S1. Full database search strategies and records identified; Supplementary Table S2. Related publications and intervention comparability assessment; Supplementary Table S3. Intervention-based classification of TMD/orofacial pain-related records and related publications; Supplementary Table S4. GCPS grades and pain-related disability categories before and after therapeutic LA injections; Supplementary Figure S1. Schematic representation of injection regions used at each session. (A) Bilateral supraorbital, infraorbital, and mental nerve exit-point regions (six injections; 0.5 mL per site). (B) Bilateral entry sites for injections in the anatomical projection area of the otic ganglion (2.5 mL per side). Shaded areas indicate muscles in which additional trigger points were identified by palpation when present; their locations and number varied between patients and sessions. (C) Bilateral intradermal wheals placed approximately 2 cm lateral to the spinous processes at C2, C3, and C4 (six injections; 0.2 mL per site).
