# Supplementary material for: Evaluation of the Therapeutic Use of Local Anesthetics in Chronic Myogenous Temporomandibular Disorders: A Pilot Study and Evidence Gap Assessment
Source: J Clin Med. 2026 Oct 19;15(20):8112. doi: 10.3390/jcm15208112 (PMC13705829; doi:10.3390/jcm15208112)
Supplement: Supplementary file 1 [file jcm-15-08112-s001.zip › jcm-4529932-supplementary.pdf]

Supplementary Material

Supplementary Table S1. Full database search strategies and records identified

Database	Search strategy	Records identified
PubMed	("Temporomandibular Joint Disorders"[Mesh] OR "Facial Pain"[Mesh] OR "Myofascial Pain Syndromes"[Mesh] OR temporomandibular disorder*[tiab] OR temporomandibular joint disorder*[tiab] OR TMD[tiab] OR craniomandibular disorder*[tiab] OR orofacial pain[tiab] OR myofascial pain[tiab] OR myalgia[tiab] OR masticatory muscle pain[tiab] OR masticatory myalgia[tiab] OR masseter pain[tiab] OR temporalis pain[tiab] OR jaw muscle pain[tiab]) AND ("Lidocaine"[Mesh] OR "Procaine"[Mesh] OR neural therapy[tiab] OR therapeutic local anesthetic injection*[tiab] OR therapeutic local anaesthetic injection*[tiab])	285
Cochrane Library	("temporomandibular disorder" OR "temporomandibular disorders" OR "temporomandibular joint disorder" OR "temporomandibular joint disorders" OR TMD OR "craniomandibular disorder" OR "craniomandibular disorders" OR "orofacial pain" OR "myofascial pain" OR myalgia OR "masticatory muscle pain" OR "masticatory myalgia" OR "masseter pain" OR "temporalis pain" OR "jaw muscle pain") AND (lidocaine OR procaine OR "neural therapy" OR "therapeutic local anesthetic injection" OR "therapeutic local anesthetic injections" OR "therapeutic local anaesthetic injection" OR "therapeutic local anaesthetic injections")	216
LIVIVO	("temporomandibular disorder" OR "temporomandibular disorders" OR "temporomandibular joint disorder" OR "temporomandibular joint disorders" OR TMD OR "craniomandibular disorder" OR "craniomandibular disorders" OR "orofacial pain" OR "myofascial pain" OR myalgia OR "masticatory muscle pain" OR "masticatory myalgia" OR "masseter pain" OR "temporalis pain" OR "jaw muscle pain") AND (lidocaine OR procaine OR "neural therapy" OR "therapeutic local anesthetic injection" OR "therapeutic local anesthetic injections" OR "therapeutic local anaesthetic injection" OR "therapeutic local anaesthetic injections")	291
Ovid MEDLINE	(exp Temporomandibular Joint Disorders/ or exp Facial Pain/ or exp Myofascial Pain Syndromes/ or (temporomandibular disorder* or temporomandibular joint disorder* or TMD or craniomandibular disorder* or orofacial pain or myofascial pain or myalgia or masticatory muscle pain or masticatory myalgia or masseter pain or temporalis pain or jaw muscle pain).ti,ab,kf.) and (exp Lidocaine/ or exp Procaine/ or (neural therapy or therapeutic local anesthetic injection* or therapeutic local anaesthetic injection*).ti,ab,kf.)	270
Scopus	TITLE-ABS(("temporomandibular disorder" OR "temporomandibular disorders" OR "temporomandibular joint disorder" OR "temporomandibular joint disorders" OR TMD OR "craniomandibular disorder" OR "craniomandibular disorders" OR "orofacial pain" OR "myofascial pain" OR myalgia OR "masticatory muscle pain" OR "masticatory myalgia" OR "masseter pain" OR "temporalis pain" OR "jaw muscle pain") AND (lidocaine OR procaine OR "neural therapy" OR "therapeutic local anesthetic injection" OR "therapeutic local anesthetic injections" OR "therapeutic local anaesthetic injection" OR "therapeutic local anaesthetic injections"))	188
Web of Science	((TI=("temporomandibular disorder" OR "temporomandibular disorders" OR "temporomandibular joint disorder" OR "temporomandibular joint disorders" OR TMD OR "craniomandibular disorder" OR "craniomandibular disorders" OR "orofacial pain" OR "myofascial pain" OR myalgia OR "masticatory muscle pain" OR "masticatory myalgia" OR "masseter pain" OR "temporalis pain" OR "jaw muscle pain") OR AB=("temporomandibular disorder" OR "temporomandibular disorders" OR "temporomandibular joint	156

Database	Search strategy	Records identified
	disorder" OR "temporomandibular joint disorders" OR TMD OR "craniomandibular disorder" OR "craniomandibular disorders" OR "orofacial pain" OR "myofascial pain" OR myalgia OR "masticatory muscle pain" OR "masticatory myalgia" OR "masseter pain" OR "temporalis pain" OR "jaw muscle pain")) AND (TI=(lidocaine OR procaine OR "neural therapy" OR "therapeutic local anesthetic injection" OR "therapeutic local anesthetic injections" OR "therapeutic local anaesthetic injection" OR "therapeutic local anaesthetic injections") OR AB=(lidocaine OR procaine OR "neural therapy" OR "therapeutic local anesthetic injection" OR "therapeutic local anesthetic injections" OR "therapeutic local anaesthetic injection" OR "therapeutic local anaesthetic injections")))	
Total		1406

Supplementary Table S2. Related publications and intervention comparability assessment

Publication title	Article type	Intervention category	Reason for non-comparability or uncertainty
A case of facial myofascial pain syndrome presenting as trigeminal neuralgia	Case report	Facial trigger-point injections and infraorbital nerve block	The case involved facial trigger-point injections and an infraorbital lidocaine block; it did not evaluate the combined injection protocol used in the present cohort.
A case series of temporomandibular disorders treated with acupuncture, occlusal splint and point injection therapy.	Case series	Combined point injection therapy	Point injections of lidocaine and steroid were given for residual tender points alongside acupuncture and splint therapy; the combined protocol used in the present cohort was not evaluated.
A Comparative Pilot Study to Evaluate the Adjunctive Role of Levosulpride with Trigger Point Injection Therapy in the Management of Myofascial Pain Syndrome of Orofacial Region.	Comparative clinical study	Trigger point injection (0.5% bupivacaine) + levosulpiride	The study evaluated isolated trigger point injection or local needling; this was considered clinically related but not directly comparable with the present therapeutic LA protocol.
A Comparative Study of Trigger Point Therapy with Local Anaesthetic (0.5 % Bupivacaine) Versus Combined Trigger Point Injection Therapy and Levosulpiride in the Management of Myofascial Pain Syndrome in the Orofacial Region.	Randomized controlled trial	Trigger point injection (0.5% bupivacaine)	The study evaluated isolated trigger point injection or local needling; this was considered clinically related but not directly comparable with the present therapeutic LA protocol.
Case report: Treatment of temporomandibular joint disorder pain with V3 nerve block procedure	Case report	V3 mandibular nerve block	A nerve or periarticular block was used, but the protocol did not correspond to the present therapeutic LA framework targeting neuroanatomical, segmental, or autonomic sites.
Comparison between Collagen and Lidocaine Intramuscular Injections in Terms of Their Efficiency in Decreasing Myofascial Pain within Masseter Muscles: A Randomized, Single-Blind Controlled Trial	Research article	Intramuscular lidocaine injection	The injection was limited to muscle tissue and did not include neuroanatomical, segmental intradermal, or autonomic/neuroregulatory targets comparable with the present protocol.
Comparison between intra-articular platelet-rich plasma injection versus hydrocortisone with local anesthetic injections in temporomandibular disorders: A double-blind study	Double-blind comparative clinical study	Intra-articular PRP vs hydrocortisone + local anesthetic	The main intervention was PRP/PRGF or steroid-based; local anesthetic was not the primary component of a comparable therapeutic LA protocol.
Dextrose prolotherapy for chronic temporomandibular pain and dysfunction: results of a pilot-level randomized controlled study	Randomized controlled trial	Dextrose prolotherapy	The primary intervention was dextrose/prolotherapy; local anesthetic, when present, was used as a comparator, carrier, or procedural component.
Dextrose Prolotherapy Versus Lidocaine Injection for	Randomized controlled trial	Dextrose prolotherapy vs lidocaine control	The primary intervention was dextrose/prolotherapy; local anesthetic, when

Publication title	Article type	Intervention category	Reason for non-comparability or uncertainty
Temporomandibular Dysfunction: A Pragmatic Randomized Controlled Trial			present, was used as a comparator, carrier, or procedural component.
Discomfort and bite force in painful masseter muscles after intramuscular injections of local anesthetic and saline solution	Controlled clinical study	Intramuscular lidocaine injection	The injection was limited to muscle tissue and did not include neuroanatomical, segmental intradermal, or autonomic/neuroregulatory targets comparable with the present protocol.
Effect of an anesthetic injected into the temporomandibular joint space in patients with TMD	Pre-post clinical study	Intra-articular TMJ lidocaine injection	The intervention was intra-articular and joint-directed rather than a therapeutic LA protocol targeting neuroanatomical, segmental, or autonomic sites.
Effect of combination of trigger point injection and stellate ganglion block on non-odontogenic mandibular molar pain referred from masseter muscle: a case report	Case report	Trigger point injection + stellate ganglion block	Although an autonomic block was included, this was a case report of non-odontogenic referred molar pain and did not use a therapeutic LA protocol comparable with the present myogenous TMD intervention.
Effects of intramuscular morphine in men and women with temporomandibular disorder with myofascial pain	Randomized double-blind clinical study	Intramuscular morphine; lidocaine comparator arm	The local anesthetic component was used as a comparator and/or intramuscular injection, not as a therapeutic LA protocol comparable with the present intervention.
Effects of occlusal treatment and intraarticular injections on temporomandibular joint pain and dysfunction	Comparative clinical study	Intra-articular corticosteroid + local anesthetic	The intervention was intra-articular and joint-directed rather than a therapeutic LA protocol targeting neuroanatomical, segmental, or autonomic sites.
Efficacy of Intra-articular Platelet-rich Plasma versus Hydrocortisone with Local Anaesthetic Injection in Temporomandibular Joint Disorders - A Prospective Study	Prospective clinical study	Intra-articular PRP vs hydrocortisone + local anesthetic	The main intervention was PRP/PRGF or steroid-based; local anesthetic was not the primary component of a comparable therapeutic LA protocol.
Efficacy of lidocaine versus mepivacaine in the management of myofascial pain	Comparative clinical study	Trigger point injection: lidocaine vs mepivacaine	The study evaluated isolated trigger point injection or local needling; this was considered clinically related but not directly comparable with the present therapeutic LA protocol.
Electromyography and bite force studies of muscular function and dysfunction in masticatory muscles	Controlled clinical study	Masseter intramuscular lidocaine injection	The injection was limited to muscle tissue and did not include neuroanatomical, segmental intradermal, or autonomic/neuroregulatory targets comparable with the present protocol.
EMG versus force relationship in painful masseter muscles before and after intramuscular anesthetics and saline injections	Research article	Masseter intramuscular lidocaine injection	The injection was limited to muscle tissue and did not include neuroanatomical, segmental intradermal, or autonomic/neuroregulatory targets comparable with the present protocol.
Evaluation of dry needling and 0.5% lidocaine injection therapies in myofascial pain trigger points in masticatory muscles	Research article	Trigger point injection (0.5% lidocaine)	The study evaluated isolated trigger point injection or local needling; this was considered clinically related but not directly comparable with the present therapeutic LA protocol.

Publication title	Article type	Intervention category	Reason for non-comparability or uncertainty
Evaluation of iontophoretically applied dexamethasone for painful pathologic temporomandibular joints.	Controlled clinical study	Iontophoresis: dexamethasone + lidocaine vehicle	The study used iontophoresis rather than an injection protocol comparable with the present therapeutic LA intervention.
Evaluation of the effects of occlusal splint and masseter muscle injection in patients with myofascial pain: a randomised controlled trial	Randomized controlled trial	Masseter lidocaine injection ± occlusal splint	The injection was limited to muscle tissue and did not include neuroanatomical, segmental intradermal, or autonomic/neuroregulatory targets comparable with the present protocol.
Evaluation of the effects of occlusal splint, trigger point injection and arthrocentesis in the treatment of internal derangement patients with myofascial pain disorders.	Comparative clinical study	Trigger point injection ± splint/arthrocentesis	The study evaluated isolated trigger point injection or local needling; this was considered clinically related but not directly comparable with the present therapeutic LA protocol.
Intra-articular platelet-rich plasma injection versus hydrocortisone with local anesthetic injections for temporo mandibular disorders	Comparative clinical study	Intra-articular PRP vs hydrocortisone + local anesthetic	The main intervention was PRP/PRGF or steroid-based; local anesthetic was not the primary component of a comparable therapeutic LA protocol.
Laser therapy and needling in myofascial trigger point deactivation	Research article	Needling + 2% lidocaine injection	The study evaluated isolated trigger point injection or local needling; this was considered clinically related but not directly comparable with the present therapeutic LA protocol.
Local injection therapy in 107 patients with myofascial pain syndrome of the head and neck	Double-blind comparative clinical study	Local injection therapy: bupivacaine/lidocaine/saline	The intervention involved local infiltration/trigger point injection and did not clearly correspond to a therapeutic LA protocol comparable with the present intervention.
Pain-pressure threshold in painful jaw muscles following trigger point injection	Controlled clinical study	Masseter trigger point local anesthetic injection	The study evaluated isolated trigger point injection or local needling; this was considered clinically related but not directly comparable with the present therapeutic LA protocol.
Plasma rich in growth factors injection effectiveness for myofascial pain treatment in masticatory muscles. Randomised controlled trial	Randomized controlled trial	PRGF vs lidocaine Trigger point injection	The study compared PRGF with lidocaine trigger point injection as a local comparator; it did not use a therapeutic LA protocol comparable with the present intervention.
Temporomandibular joint injection with corticosteroid and local anesthetic for limited mouth opening	Retrospective study	Intra-articular corticosteroid + local anesthetic	The intervention was intra-articular and joint-directed rather than a therapeutic LA protocol targeting neuroanatomical, segmental, or autonomic sites.
Temporomandibular joint iontophoresis: a double-blind randomized clinical trial	Randomized double-blind clinical study	Iontophoresis: dexamethasone + lidocaine	The study used iontophoresis rather than an injection protocol comparable with the present therapeutic LA intervention.
The effectiveness of physical factors in the treatment of compression-dislocation dysfunction of the temporomandibular joint.	Comparative clinical study	Periarticular lidocaine block vs physical therapy	A nerve or periarticular block was used, but the protocol did not correspond to the present therapeutic LA framework targeting neuroanatomical, segmental, or autonomic sites.

Publication title	Article type	Intervention category	Reason for non-comparability or uncertainty
The efficacy of dry needling and procaine in the treatment of myofascial pain in the jaw muscles	Randomized double-blind clinical study	Procaine injection vs dry needling	The study evaluated isolated trigger point injection or local needling; this was considered clinically related but not directly comparable with the present therapeutic LA protocol.
Treatment of an acute anterior disk displacement in the temporomandibular joint. A case report	Case report	Iontophoresis + home exercise	The study used iontophoresis rather than an injection protocol comparable with the present therapeutic LA intervention.
Treatment of Temporomandibular Dysfunction With Hypertonic Dextrose Injection (Prolotherapy): A Randomized Controlled Trial With Long-term Partial Crossover	Randomized controlled trial	Intra-articular dextrose/lidocaine versus intra-articular lidocaine	Lidocaine was included in the comparator arm, but both treatments were joint-directed rather than the combined injection protocol used in the present cohort.
Trigger point injection therapy in the management of myofascial temporomandibular pain.	Comparative clinical study	Trigger point injection + stabilization splint	The study evaluated isolated trigger point injection or local needling; this was considered clinically related but not directly comparable with the present therapeutic LA protocol.
Trigger point injection: a neglected modality in the treatment of TMJ dysfunction	Unclear (full text unavailable)	Reported trigger-point injection; details unavailable	An abstract and full text were unavailable to verify the injection sites or determine intervention comparability.
Ultrasound-Guided Vs Non-Guided Prolotherapy for Internal Derangement of Temporomandibular Joint. A Randomized Clinical Trial.	Research article	Dextrose prolotherapy with intra-articular and masseter injections	The intervention included a joint injection and an injection into a tender point in the masseter muscle, but did not evaluate the combined protocol used in the present cohort.

Supplementary Table S3. Intervention-based classification of TMD/orofacial pain-related records and related publications

Intervention category	Number of records	Reason for non-comparability or uncertainty
**A. Studies not carried forward after Step 4 (*n*=20)**		
Arthrocentesis / joint lavage	6	Joint-directed procedure; not comparable with the therapeutic LA protocol used in the present study.
Prolotherapy / dextrose injection	3	The primary intervention was dextrose/prolotherapy rather than a therapeutic LA protocol.
Botulinum toxin injection	3	Pharmacologically different intervention; not a therapeutic LA protocol.
Intra-articular injections not consistent with the present intervention framework	3	Joint-directed intervention without neuroanatomical, segmental intradermal, or autonomic/neuroregulatory targets comparable with the present protocol.
Hyaluronic acid / sodium hyaluronate injections	2	Primary intervention was viscosupplementation or joint-directed treatment.
Ozone injection	1	Primary intervention was ozone rather than therapeutic LA.
Unclear or insufficiently defined intervention	2	Intervention details could not be verified; comparability remains uncertain.
**Subtotal**	**20**	**Not carried forward for intervention comparability assessment.**
**B. Related publications assessed for intervention comparability (*n*=36)**		
Trigger point injection / local needling alone	11	Clinically related, but isolated trigger point injection/local needling alone was not directly comparable with the present therapeutic LA protocol.
Isolated intramuscular local anesthetic injection	6	Local anesthetic was injected into masticatory muscles without neuroanatomical, segmental intradermal, or autonomic/neuroregulatory targets comparable with the present protocol.
PRP/PRGF or steroid comparison studies	3	Local anesthetic was not the primary therapeutic component of a comparable LA protocol.
Prolotherapy / dextrose studies	4	The primary intervention was dextrose/prolotherapy rather than therapeutic LA.
Iontophoresis studies	3	Local anesthetic was delivered topically or electrically rather than by an injection protocol comparable with the present intervention.
Nerve / periarticular block without a comparable therapeutic LA protocol	2	A block technique was used, but the protocol did not correspond to the present therapeutic LA framework.
Intra-articular corticosteroid/local anesthetic studies	2	Joint-directed intra-articular intervention; not directly comparable with the present therapeutic LA protocol.
Other borderline or unclear interventions	5	These records involved different or mixed interventions, different target populations, or insufficient information to determine comparability.
**Subtotal**	**36**	No study of the complete protocol was identified among publications with sufficient intervention detail; comparability remained uncertain where details were insufficient.
**Overall total**	**56**	**20 records not carried forward and 36 related publications.**

PRGF: plasma rich in growth factors; PRP: platelet-rich plasma; TMD: temporomandibular disorder.

Supplementary Table S4. GCPS grades and pain-related disability categories before and after therapeutic LA injections

Outcome	Category	Baseline *n* (%)	Follow-up *n* (%)
GCPS grade	0	6 (22.2%)	18 (66.7%)
GCPS grade	1	10 (37.0%)	4 (14.8%)
GCPS grade	2	3 (11.1%)	4 (14.8%)
GCPS grade	3	7 (25.9%)	1 (3.7%)
GCPS grade	4	1 (3.7%)	0 (0%)
Pain-related disability category	None	6 (22.2%)	18 (66.7%)
Pain-related disability category	No disability	13 (48.1%)	8 (29.6%)
Pain-related disability category	Moderately limiting	7 (25.9%)	1 (3.7%)
Pain-related disability category	Severely limiting	1 (3.7%)	0 (0%)

GCPS: Graded Chronic Pain Scale; LA: local anesthetic. In the main text, these data are summarized narratively to reduce table burden.

Wilcoxon signed-rank test: GCPS grades p=0.006; pain-related disability categories p=0.003.

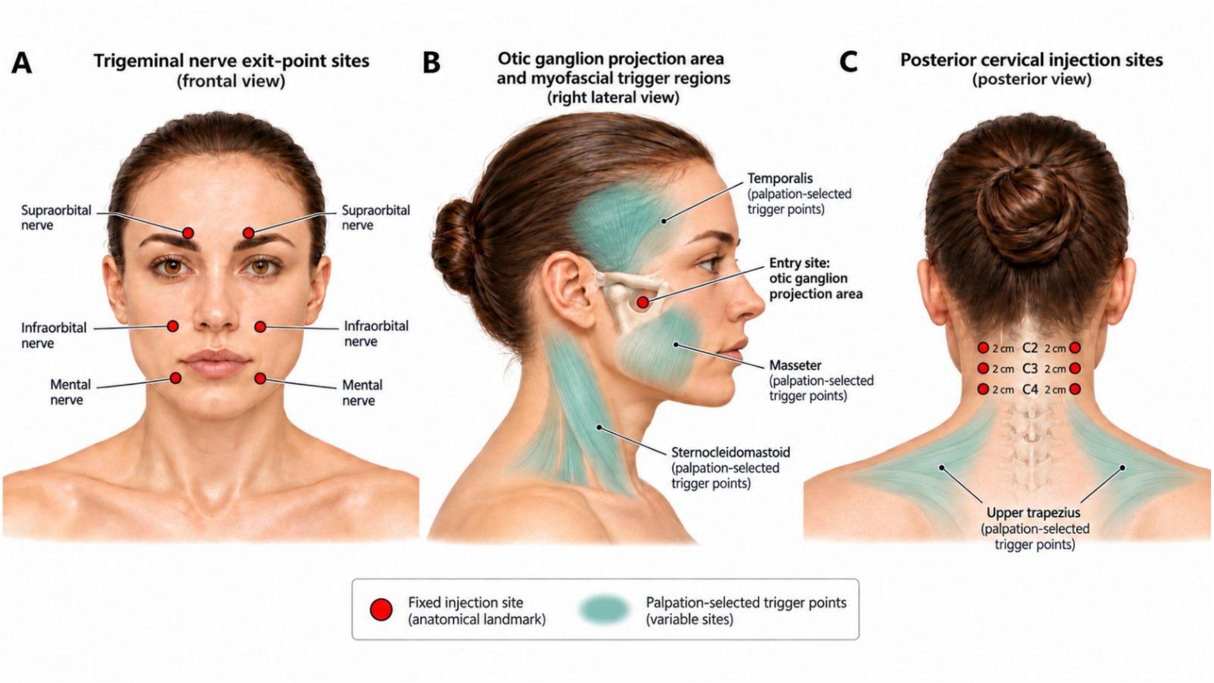

Supplementary Figure S1. Schematic representation of injection regions used at each session. (A) Bilateral supraorbital, infraorbital, and mental nerve exit-point regions (six injections; 0.5 mL per site). (B) Bilateral entry sites for injections in the anatomical projection area of the otic ganglion (2.5 mL per side). Shaded areas indicate muscles in which additional trigger points were identified by palpation when present; their locations and number varied between patients and sessions. (C) Bilateral intradermal wheals placed approximately 2 cm lateral to the spinous processes at C2, C3, and C4 (six injections; 0.2 mL per site).
